# A Decentralized ComBat Algorithm and Applications to Functional Network Connectivity

**DOI:** 10.3389/fneur.2022.826734

**Published:** 2022-03-15

**Authors:** Biozid Bostami, Frank G. Hillary, Harm Jan van der Horn, Joukje van der Naalt, Vince D. Calhoun, Victor M. Vergara

**Affiliations:** ^1^Department of Computer Science, Tri-institutional Center for Translational Research in Neuroimaging and Data Science, Georgia State University, Atlanta, GA, United States; ^2^Department of Computer Science, Georgia Institute of Technology, Atlanta, GA, United States; ^3^Department of Computer Science, Emory University, Atlanta, GA, United States; ^4^Department of Psychology, Penn State University, State College, PA, United States; ^5^Department of Developmental Neurology, University of Groningen, University Medical Center Groningen, Groningen, Netherlands; ^6^Department of Medical Sciences, University of Groningen, University Medical Center Groningen, Groningen, Netherlands

**Keywords:** harmonization, federated learning, neuroimage analysis, functional connectivity, brain network

## Abstract

Recent studies showed that working with neuroimage data collected from different research facilities or locations may incur additional source dependency, affecting the overall statistical power. This problem can be mitigated with data harmonization approaches. Recently, the ComBat method has become commonly adopted for various neuroimage modalities. While open neuroimaging datasets are becoming more common, a substantial amount of data is still unable to be shared for various reasons. In addition, current approaches require moving all the data to a central location, which requires additional resources and creates redundant copies of the same datasets. To address these issues, we propose a decentralized harmonization approach that does not create redundant copies of the original datasets and performs remote operations on the datasets separately without sharing any individual subject data, ensuring a certain level of privacy and reducing regulatory hurdles. We proposed a novel approach called “Decentralized ComBat” which can harmonize datasets separately without combining the datasets. We tested our model by harmonizing functional network connectivity datasets from two traumatic brain injury studies in a decentralized way. Also, we used simulations to analyze the performance and scalability of our model when the number of data collection sites increases. We compare the output with centralized ComBat and show that the proposed approach produces similar results, increasing the sensitivity of the functional network connectivity analysis and validating our approach. Simulations show that our model can be easily scaled to many more datasets based on the requirement. In sum, we believe this provides a powerful tool, further complementing open data and allowing for integrating public and private datasets.

## Introduction

The significance of network neuroscience has reached a global scale with a growing number of large-scale projects related to impactful topics such as brain disease, brain development, brain aging, and brain-computer interfacing (1–3). Maximizing the potential of these large projects to reach their goal depends on the data at one's disposal, which urges global collaboration, knowledge, and data sharing. These collaborative approaches include aggregating data collection to a central repository or data sharing based on data usage agreements (DUA) (4, 5). Such an approach has several limitations to consider. The first concern is the policy and proprietary restrictions, or data de-identification issues may be raised. Such concerns are time-consuming and take months to resolve.

Moreover, the processing of DUA can consume a large amount of time. Another significant concern is the volume of the data collected from multiple sites because merging large neuroimage datasets in a single location consumes redundant space. Additionally, computational resources become costly when the volume of data grows. Also, sharing the data only creates redundant copies around the world. Thus, it is not always an optimal approach considering the constraints on available resources. While open neuroimage datasets are becoming more common, some data cannot be transferred or shared directly due to confidentiality or regulatory constraints. These issues led to a paradigm shift toward decentralized data-sharing (6, 7) which is particularly true with widespread efforts in the neuroimage community to maximize study power through multi-site investigation, data sharing, and team science.

With the availability of neuroimage data at multiple sites worldwide, an important goal is to jointly analyze geographically dispersed data to increase statistical power and test against the common biological hypothesis. There is an issue with combining the multi-site neuroimage data because each data at a different location introduces additional non-biological variability. These variabilities are closely related to image acquisition protocol and scanner parameters categorized as “site effects” (8). These site effects can reduce statistical power or lead to erroneous conclusions. Harmonization techniques aim to combine datasets generated from different sites, e.g., hospitals, research facilities, or laboratories, reducing the site effects in the combined dataset (9).

One popular harmonization technique is known as ComBat (10). The ComBat technique was first introduced in genomics to reduce batch effects and non-biological variability due to pooling batches of sample genes from various laboratories. Later, it was applied to diffusion tensor imaging (DTI) (9), cortical thickness data (11), functional connectivity measures (12), Dynamic Functional Network Connectivity (13). However, the current ComBat model does not address data access problems, including geographical and confidentiality issues, which motivate us to develop a decentralized model that works in a distributed environment. This manuscript presents a decentralized harmonization model called “Decentralized ComBat (DC-ComBat)”.

For several years, our team has been working on a web-based framework to analyze data stored in multiple locations without pooling named Collaborative Informatics and Neuroimaging Suite Toolkit for Anonymous Computation (COINSTAC) (14). This framework also preserves the privacy of the data as there is no data pooling involved and all the communication between the sites is encrypted. COINSTAC uses a message-passing infrastructure to implement decentralized algorithms to work with geographically scattered datasets. We can develop a decentralized algorithm that returns similar results on collected datasets with this framework. This framework preserves dataset privacy by not creating additional copies. Also, this framework can be scaled easily when the number of sites or datasets increases. There are several decentralized algorithms already implemented using COINSTAC. Some of the decentralized computation proposed earlier include decentralized regression (14), decentralized temporal independent component analysis (15), decentralized independent vector analysis (16), decentralized neural networks (17), decentralized data ICA (18), decentralized PCA (19), and many more. Some of these algorithms can be used jointly with our decentralized harmonization approach in the COINSTAC for creating different pipelines. We found this framework suitable for our decentralized approach based on the benefits.

## Methods

ComBat can be described as follows if the data is collected from ***k*** different sites where each site has **n**_**i**_ scans where ***i*** = 1, 2, …, ***k***. Each harmonized feature **y** indexed by ***v*** of scan ***j*** at site ***i***, the value **y**_**i**, **j, v**_ can be defined as:


(1) $$\begin{array}{r} y_{i,j,v}=\alpha_{v}+X_{i,j}\beta_{v\;}+\gamma_{i,v}+\delta_{i,v}\varepsilon_{i,j,v} \end{array}$$

In the above equation **α**_**v**_ represents the overall mean value at feature **ν**. ***X*** represents the biological variants, **β**_**ν**_ represents the regression coefficient for **X** at feature **ν**. The error term **ε** is assumed to follow a Gaussian distribution **N(0,σ**^**2**^**)**. In Equation (1) **δ**_**i**, **ν**_ and **γ**_**i**, **ν**_ represents the multiplicative and additive parameters correcting for site effects at site ***i*** for feature ***v***. The model aims to reduce the unwanted variance using the Empirical Bayes approach. The final distribution model can be achieved by:


(2) $$\begin{array}{r} y_{ij\nu }^{comBat}=\frac{y_{ij\nu }-{\hat{\alpha }}_{\nu }-X_{ij}{\hat{\beta }}_{\nu }-{\hat{\gamma }}_{i\nu }}{{\hat{\delta }}_{i\nu }}+{\hat{\alpha }}_{\nu }+X_{ij}{\hat{\beta }}_{\nu } \end{array}$$

The model can be divided into three parts. The first part is the standardization of data. The Decentralized regression algorithm available in COINSTAC (14) was used to calculate the initial β-coefficients. We calculated the local mean and local variance based on β-coefficients in later stages. After standardization, every data will have similar overall mean and variance. The following equation calculates the standardization data:


(3) $$\begin{array}{r} Z_{i,j,\nu }=\frac{y_{ij\nu }-{\hat{\alpha }}_{\nu }-X_{ij}{\hat{\beta }}_{\nu }}{{\hat{\sigma }}_{\nu }} \end{array}$$

The second part is the estimation of batch effect using parametric empirical priors. The ComBat assumes that the standardized data **Z**_**i, j, v**_ follows the standard distribution form, $Z_{i,j,v}\;\sim \;N\left(\gamma_{i,v},\;{\text{δ}}_{i,v}^{2}\right)$. It is also mentioned that parametric forms of the prior distributions on the batch effect parameters, $\gamma_{i,v},\;{\text{δ}}_{i,v}^{2}$ follows a normal distribution and Inverse gamma distribution, respectively. Defined by:


(4) $$\begin{array}{r} \gamma_{i,\nu }\;\sim \;N\left(Y_{i},\;\tau_{i}^{2}\right)\text{ and }\delta_{i,\nu }^{2}\;\sim \text{ Inverse Gamma}\left(\lambda_{i},\theta_{i}\right) \end{array}$$

The hyperparameters $\gamma_{\text{i}},\tau_{\text{i}}^{2},\lambda_{\text{i}},\theta_{\text{i}}$ are estimated empirically from the standardized data. Details of the derivation of the estimators are explained in the Supplementary Material of the original ComBat paper (8). Based on the Empirical Bayes estimators $\gamma_{i,v},\;\delta_{i,\nu }^{2}$ can be defined by the posteriors means as followings:


(5) $$\begin{array}{r} \gamma_{i,\nu }^{{}^{*}}\;=\;\frac{n_{i}\tau_{i}^{2}\hat{\gamma_{i,\nu }}+\delta_{i,\nu }^{2^{*}}{\bar{\gamma }}_{i}}{n_{i}\tau_{i}^{2}+\delta_{i,\nu }^{2^{*}}}\text{ and }\delta_{i,\nu }^{2^{*}}\;=\;\frac{{\bar{\theta }}_{i}+\;\frac{1}{2}\underset{j}{\sum }{\left(Z_{ij\nu }-\gamma_{i,\nu }^{{}^{*}}\;\right)}^{2}}{n_{i}\tau_{i}^{2}+\delta_{i,\nu }^{2^{*}}} \end{array}$$

Finally, data is adjusted based on the estimated site parameters $\gamma_{i,v}^{*}$ *and* ${\text{δ}}_{i,v}^{2^{*}}$.

The described ComBat model does not address working in a decentralized environment. We proposed a decentralized model that can operate on separate datasets and produce identical results to the original model. We implemented the decentralized ComBat (DC-ComBat) using a platform COINSTAC. The architecture of DC-ComBat- is discussed in the following section.

## Decentralized ComBat Model Overview

In our decentralized environment, we have two types of nodes: The first type is the aggregator node, also known as the remote node which does not hold any data and acts as a storage of intermediate results and performs simple operations such as aggregation. The second node type is the local/regional node where datasets are located. These local nodes represent the participants who are willing to collaboratively. With the help of COINSTAC, we created a network where the regional nodes can be connected to the remote node and perform different operations synchronously.

**Figure 1:**
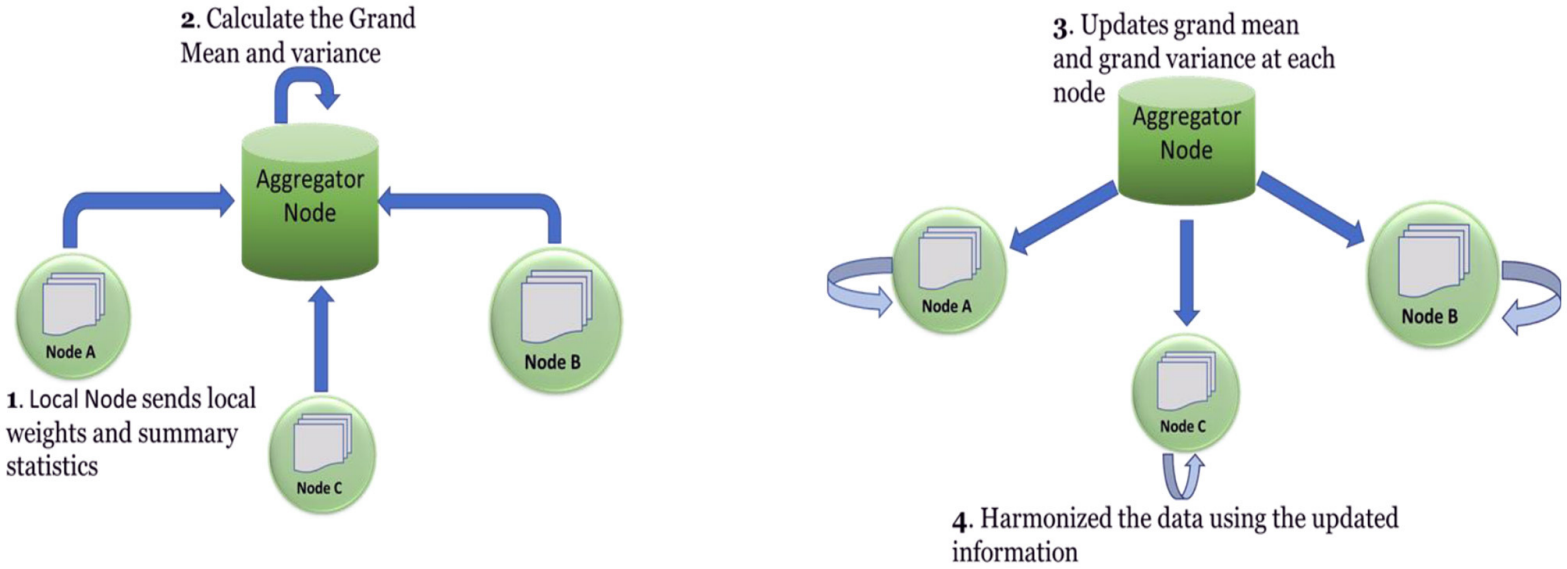
Gives the overall picture of the decentralized ComBat algorithm and intra-communication between nodes.

For harmonizing distributed datasets located at different locations, we first constructed a network prototype shown in Figure 1 where all the participating local nodes connect with the remote node. Then each participating local node shares the local weights and summary statistics with the remote node via the secured message-passing mechanism [Figure 1(1)]. All intermediate communication is encrypted and sent over TLS (Transport Layer Security) provided by COINSTAC (14). The remote node calculates the grand mean and grand variance by aggregating the regional nodes' values in Figure 1(2) and broadcasting the grand mean and grand variance to all local nodes in Figure 1(3). After receiving the grand mean and grand variance information from the remote node, each node performs data standardization on the dataset located at each node [Figure 1(4)]. Following the data standardization, estimation of site effect using parametric empirical priors is done on each site. Moreover, each site can adjust and harmonize the local data concerning the other participating site nodes based on the estimated site parameters. The pseudo algorithm is given below:

**Table 1:** **Algorithm**:

**Step1**: Initialize tde central node and site nodes.
**Step2**: Collect tde initial summary (number of samples) of tde site nodes in tde central node.
**Step3**: Calculate tde β coefficient for each site using tde decentralized regression approach available in COINSTAC.
**Step4**: For site node i = 1, 2, 3 …. N do
1. calculate tde local mean across tde features using tde local β coefficients
2. calculate tde local variance across tde feature using tde local β coefficients
3. send tde local mean and variance to tde aggregator node.
4. end for loop.
**Step5**: compute grand mean and grand variance and update each site node.
**Step6:** For site node i = 1, 2, 3 …. N do
1. standardized tde data w.r.t tde grand mean and grand variance.
2. estimate tde site parameters $\gamma_{i,v}{}^{*}and\;\delta_{i,v}^{2^{*}}$.
3. Adjust tde data accordingly.
4. Save tde adjusted data.
5. end for loop.

## Data Collection and Pre-processing

We used two sets of data for experimenting with our decentralized ComBat model. The first set consists of static FNC (functional network connectivity) data collected from two studies on mild traumatic brain injuries (mTBI) (20). We wanted to observe the performance of our model when it is applied to FNC data as from the previous study presented in (10), which showed that the ComBat model performs well on removing site effects from FNC datasets. The second set consists of simulated data generated using a connectivity template. The second dataset was used to measure the performance and scalability of our model when the number of sources increases. We tried to simulate a real-world situation where datasets are located at different locations across the world can be harmonized simultaneously. In the following sections, we will describe how these two sets of datasets were collected and pre-processed.

### Dataset 1

This dataset consists of data collected from two cohorts. The first cohort was collected from New Mexico (NM). All participants provided informed consent according to the Declaration of Helsinki and the institutional review board guidelines at the University of New Mexico. The second cohort was collected from the Netherlands Europe (EU). The local Medical Ethics Committee of the UMCG approved the data collection protocol, and every participant provided written informed consent. All procedures were conducted following the declaration of Helsinki. This data was also used in other studies related to dynamic functional connectivity (20) and brain modalities (21). Data pre-processing and analysis were the same as described in the earlier research publication (22); therefore, we present a brief outline of the whole process.

#### New Mexico Cohort Imaging Protocol

In the New Mexico cohort, the total number of participants was 96, among which 48 were mTBI patients and 48 were healthy control (HC). The subjects had a mean age of 27.3 ± 9.0 years. The scanner used in the New Mexico cohort was a 3 Tesla Siemens TIM Trio scanner. Every participant had gone through 5 min resting state-run. TR (Repetition Time) = 2,000 ms; TE (Time of Echo) = 29 ms; flip angle = 75°; FOV (Field of View) = 240 mm; matrix size = 64 × 64. After removing the first five images due to the T1 equilibrium effect, the final 145 images were selected next step analysis.

#### Netherland (European) Cohort Imaging Protocol

In the case of the European cohort total of 74 participants were studied. There were 54 patients with mTBI and 20 Healthy controls among the participants. The mean age was 37, ranging from 19 to 64. The 3.0 T Philips Integra MRI scanner was used to collect the brain images for this group of participants. The duration was 10 min for the Netherlands cohort. TR (Repetition Time) = 2,000 ms; TE (Time of Echo) = 20 ms; flip angle = 8°; FOV (Field of View) = 224 × 224 × 136.5 mm.

#### fMRI Pre-processing

First, the fMRI data underwent Statistical Parametric Mapping (SPM) (23) and was transformed into Montreal Neurological Institute standard space. AFNI v17.1.03 software was used for de-spiking. The time courses were made orthogonal to (1) linear, quadratic, and cubic trends, (2) 6 realignment parameters, (3) derivatives of realignment parameters. Data collected from the NM participants were used in the group independent component analysis (ICA) (24) using the GIFT software (25) to gather a set of functionally independent components. For Netherland cohort data, the group information guided ICA (26) (GIGICA) algorithm was used to match the 48 selected components. Finally, discarding the artifactual components, only 48 noise-free components were chosen as resting-state networks (RSNs) for further study.

### Dataset 2

For this set, we generated data using computer simulation. The primary purpose of using a simulated dataset was to observe the scalability and performance of our model. Additionally, we used simulated data because the original ComBat model assumes that two site parameters: multiplicative and additive parameters drawn from the dataset will follow inverse-gamma and gaussian distribution. However, in practice such an assumption may not always hold. That is why we created a simulation where datasets may follow some other distribution, e.g., sub-gaussian distribution, super-Gaussian distribution, or a skewed distribution for additive parameter and Poisson, Rayleigh, or Weibull distribution for multiplicative parameter. To generate the datasets, we used an FNC (functional network connectivity) template based on an FNC matrix from a previous study (27) as the ground truth. We created various datasets by randomly adding site variance complying with the assumed normal and inverse gamma distributions. We fixed the Gaussian distribution parameters with the mean at 0.05 and the standard deviation at 0.3. For the inverse gamma distribution, we set the mean at 0.3 and the standard deviation at 0.5. We used this dataset to observe the performance of the DC-ComBat model.

## Experiment Setup 1 and Investigation

**Figure 2:**
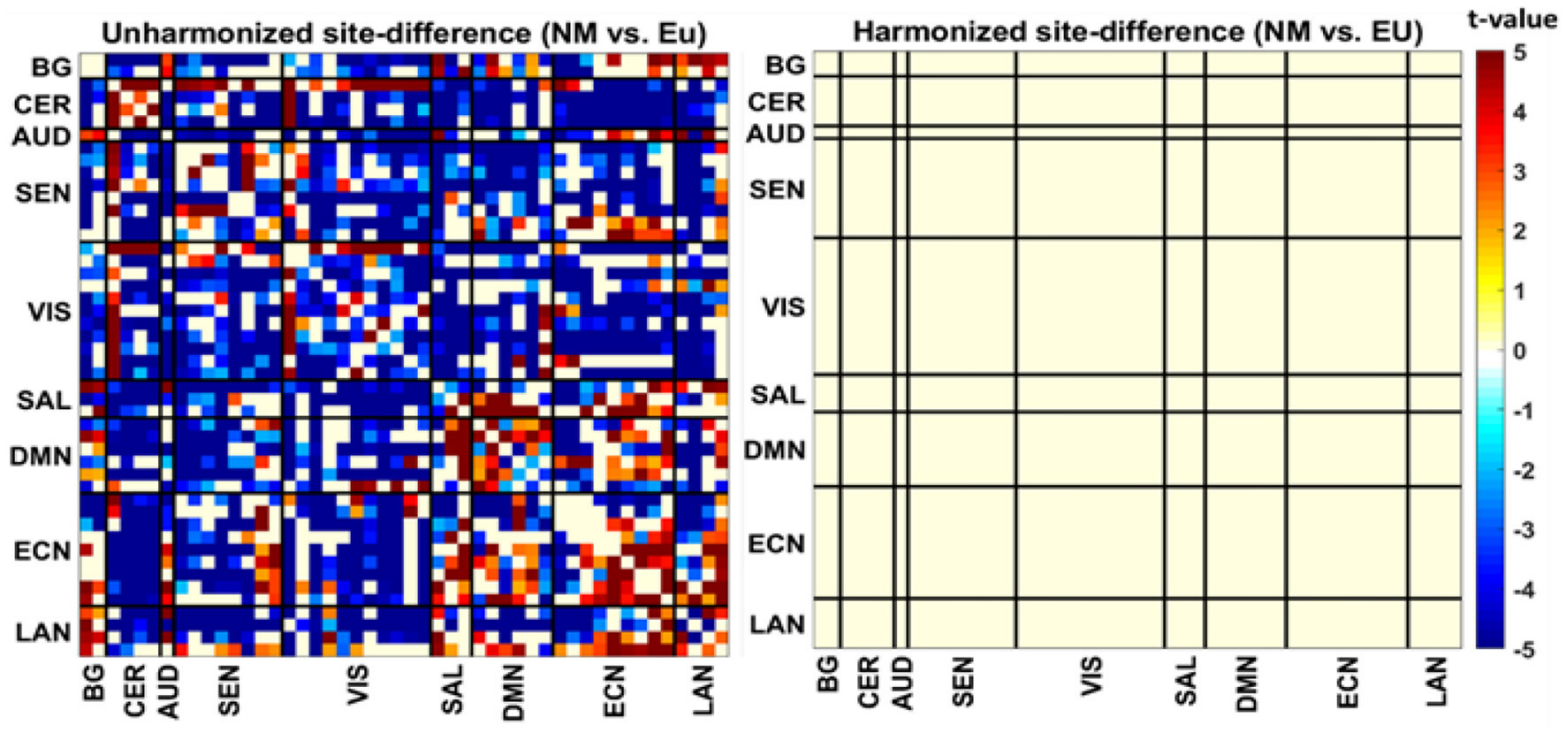
Heatmap of *t*-values site-difference (NM-EU) before **(left)** and after harmonization **(right)**.

For this experiment, we keep two datasets collected from two research facilities into two local nodes. We applied our model DC-ComBat to harmonize the datasets. To observe the harmonization performance, we perform two different assessments on the dataset. First, we compare the site differences before and after harmonization. So, we took the difference between the functional connectivity values of New Mexico (NM) and European (EU) sites, resulting in 1,128 *t*-values. Instead of showing vectors, we converted them into a matrix where rows and columns represent each of the 48 ICA components and heatmap indicates the strength of the site difference. Figure 2 shows the site difference before and after harmonization. There were a high number of significant site differences before harmonization, observed in Figure 2 (left). These indicate that site information was adding non-biological variance in the datasets, which is undesirable. After harmonization, we observed from Figure 2 (right) that all the significant site differences were removed from the data. Removal of site differences indicates a high performance of DC-ComBat.

**Figure 3:**
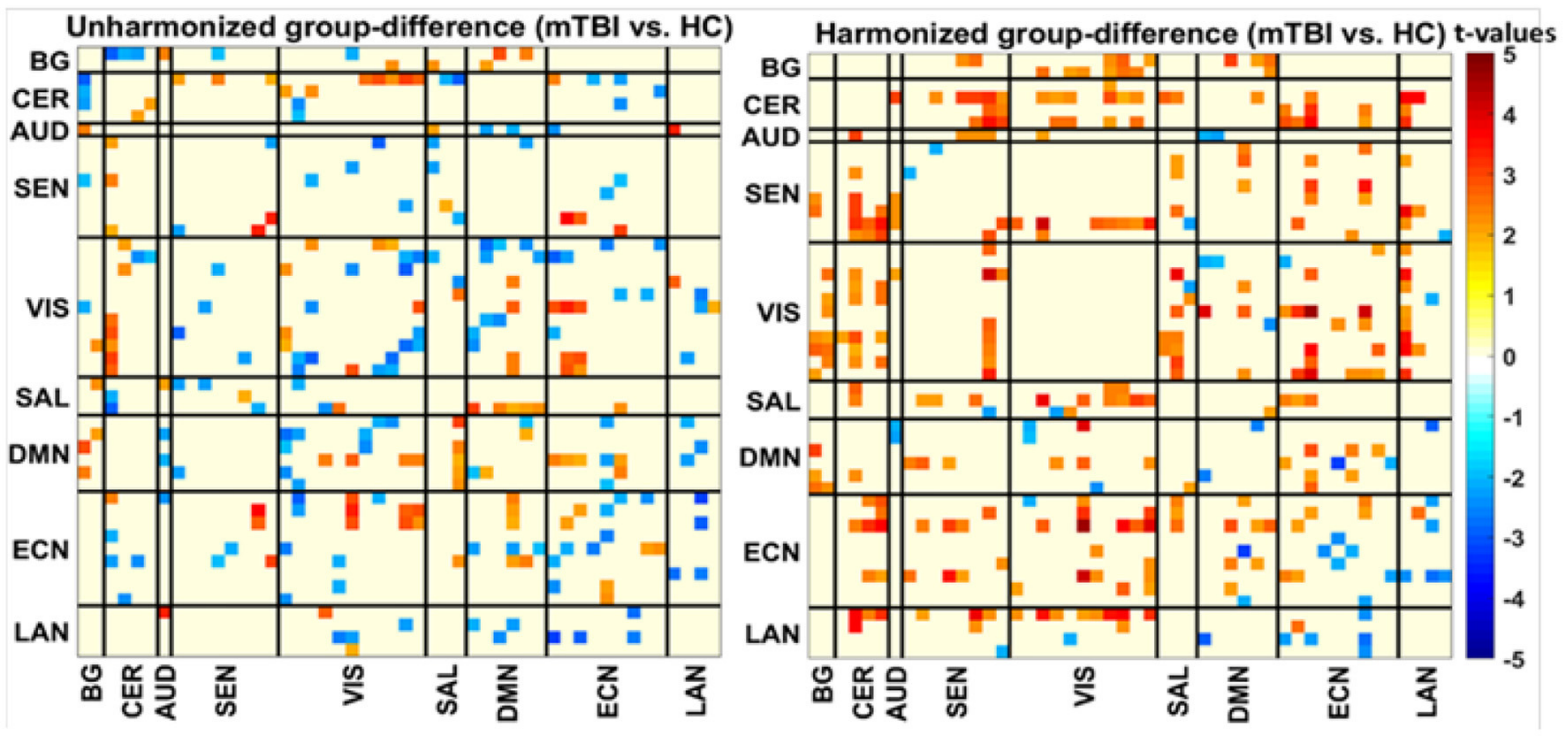
Heatmap of *t*-values group difference (NM-EU) before **(left)** and after harmonization **(right)**.

Later, we calculate the group difference (mTBI vs. HC) for the second assessment before and after harmonization. We first combined the datasets and calculated the group difference between participant groups ( mTBI and Healthy Controls) before and after harmonization. Again, based on the *t*-values, we plot the heatmap shown in Figure 3. Before harmonization, there were 128 significant *t*-values (*p* < 0.05) shown in Figure 3 (left); however, the number increased to 159 significant *t*-values when datasets were harmonized in Figure 3 (right). We observed that after harmonization, higher connectivity was observed in the TBI group in general. We observed the increase in connectivity because due to harmonization, site effects were posteriorly removed by DC-ComBat.

Furthermore, by comparing the output of the proposed decentralized ComBat with centralized ComBat, found that the maximum difference was 3.06699*e*^−15^. This very slight difference in the output was within the order of magnitude of the machine precision error. We conclude there was no effective difference between ComBat and DC-ComBat.

## Experiment 2 and Investigation

**Figure 4:**
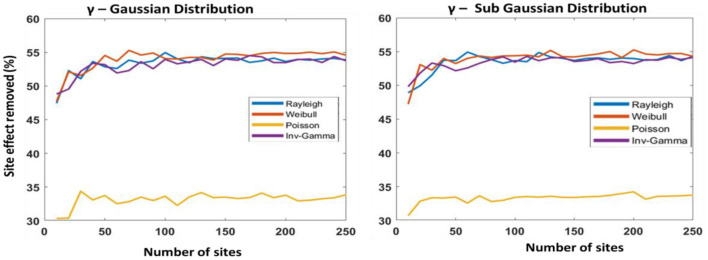
Decentralized ComBat with different distributions as the multiplicative parameter; Gaussian distribution (skewness: 0.26 and kurtosis: 3.3) **(left)** and Sub-Gaussian distribution (skewness: 0.12 and kurtosis: 2.2) **(right)** for additive parameter.

**Figure 5:**
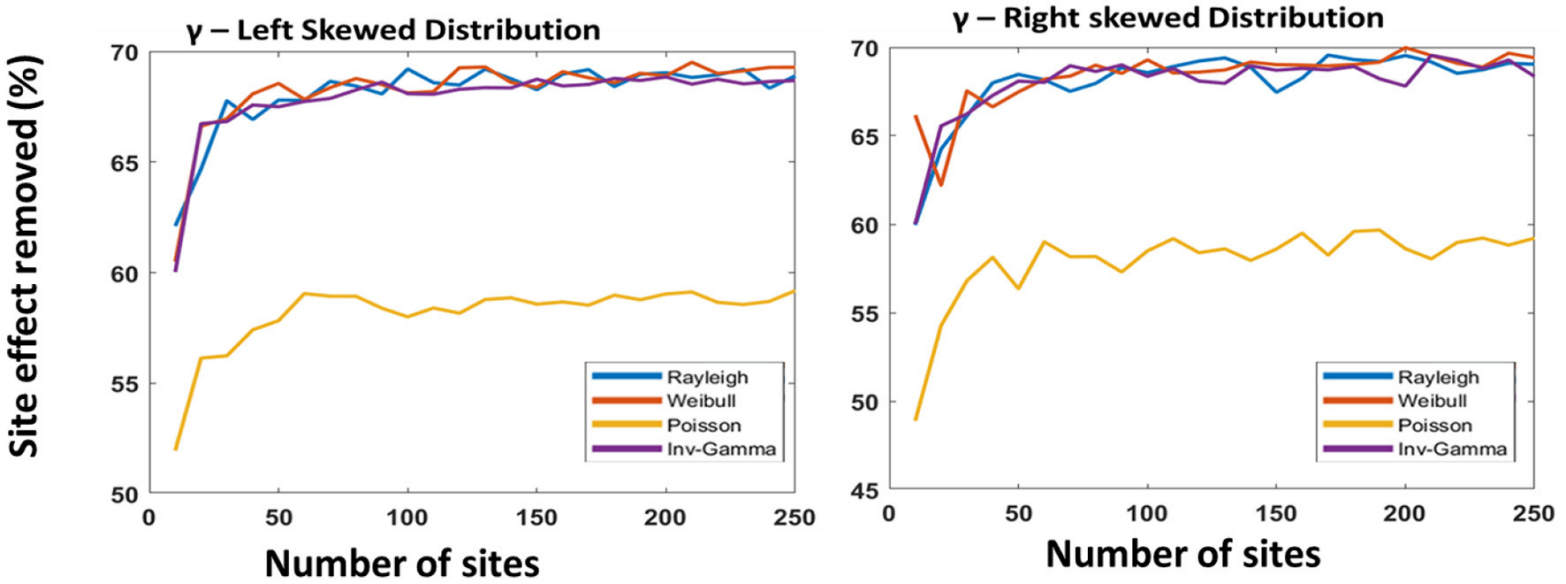
Decentralized ComBat with different distributions as the multiplicative parameter; Skewed-left distribution(skewness: −0.58 and kurtosis: 3.48) **(left)** and right skewed distribution (skewness: 0.34 and kurtosis: 2.34) **(right)** for additive parameter.

For the second experiment, we used simulation to generate data based on a functional connectivity template used as ground truth for further analysis (27). We had selected four probability distributions: Rayleigh, Weibull, Poisson, and inverse-gamma to simulate the multiplicative parameter and added noise to the ground truth. Similarly, we selected Gaussian, Sub-gaussian, Right-skewed, and Left-skewed distributions for simulating additive site parameters and added noise to the ground truth. The selection of these probability distributions was random and without any prior knowledge. After adding the noise to the ground truth, we created several datasets. We had created 250 datasets, each with 100 participants, random patients, and healthy controls. In the next step, we used COINSTAC-simulator to set the environment where each local node will contain a single dataset. Finally, we run our DC-ComBat algorithm to harmonize the datasets. We repeated the experiment by incrementing the number of sites and calculating the percentage of site effects removed with respect to the ground truth. The whole process was repeated four times by generating data with different distributions. We finally generated four plots in Figures 4, 5.

The primary purpose of this experiment was to evaluate the consistency of our model when the number of sites increases and randomness is introduced. From Figures 4, 5, we observed that our model performance was not affected when the number of sites was more than 50. We did not observe any performance issues even when the number of sites increased, indicating that our proposed model is scalable and robust.

The second purpose of using simulated data was to observe the performance of DC-ComBat when exposed to different site parameters drawn from different probability distributions. From Figures 4, 5, we observed that the skewness and kurtosis of the additive parameter affect the performance of the harmonization process. In Figure 5, we observed that when skewness and kurtosis increased, the algorithm could remove up to a maximum of 70% compared to Figure 4 where skewness and kurtosis were lower, and accuracy maximum accuracy was only 54%. We also observed from our experiment that the performance of DC-ComBat degrades for a certain distribution choice for multiplicative parameters. In Figures 4, 5, we saw that for Poisson distribution, performance is poor compared to other distributions.

## Discussion

In our work, we proposed a scalable decentralized version of ComBat which can be used for harmonizing neuroimage datasets in a decentralized fashion. From the algorithm presented above, we can observe that our model only shares simple meta-information about datasets which helps each site harmonize its dataset independently with respect to other participating sites. Also, no complex operation was performed in the remote node, so it does not require high computational power. This model has several advantages. First, data sharing becomes more manageable as it does not require the dataset transfer away from the original location. Secondly, we do not need to create redundant copies of the datasets by pooling them on a single location, saving much space and reducing the computational cost associated. Thirdly, our model can be easily extended when the number of participating sites increases. Fourthly, each node harmonizes its dataset independently which requires less computational power. Fifthly, our model is integrated with COINSTAC which provides additional security during information exchange off the shelf. Finally, we can easily combine our model with other decentralized algorithms provided by COINSTAC to create different analysis pipelines. Another main contribution of our work is that there is no significant difference between the computer parameters of centralized ComBat and decentralized ComBat.

We presented a simple star network model which could harmonize data in a decentralized environment. Also, from Figure 1, it can be observed that original data never leaves the sites, which protects the confidentiality of the datasets. Also, the computational cost is divided among the local nodes.

We observed the influence of site effects in the dataset before and after harmonization. We observed increased connectivity among the mTBI groups after harmonization because harmonization removed the site effects. Moreover, results in post-harmonized data, Figure 3 suggest that mTBI patients develop hyperconnectivity after TBI injuries. Based on the literature, increased connectivity is a regular observation in TBI as the brain reacts to the traumatic injury event (22, 28, 29). In our case, after we remove the site effects from the datasets, we observe more connectivity in the TBI groups not observed before as it was mixed with site effects. Based on the observations, we can say that harmonization does help in removing confounding non-biological effects allowing for more meaningful discoveries.

In our study, we showed that our proposed model could handle an increased number of sites. Based on the simulation, we showed that DC-ComBat could harmonize even 250 sites simultaneously. We showed in Figures 4, 5 that after the number of sites reached above 50, there was no change in performance. Moreover, the remote node does not perform any complex operation. Instead, all the complex operation such as harmonization is done on each local node. That is why the model can scale quickly when the number of participating nodes increases.

In our study, we observed that the performance of DC-ComBat is dependent on the two site parameters called additive parameter and multiplicative parameter. The base assumption of the ComBat model is that the multiplicate parameter will follow the inverse-gamma distribution, and the additive parameter will follow the Gaussian distribution. However, we cannot control the probability distributions of site parameters directly. That is why for some distributions, our proposed model may perform poorly for the Poisson distribution shown in Figures 4, 5. We observed that Rayleigh and Weibull distributions were similar to the inverse-gamma because they conjugate prior to inverse-gamma^1^, whereas Poisson is not for the inverse-gamma distribution. Moreover, we also observed that skewness and kurtosis could increase or decrease the performance of our model. We will not discuss the effects of probability distributions of site effects as it is not fully understood and will be a part of our future research direction.

The main contribution of this work is the decentralization of the harmonization process using ComBat and COINSTAC. The output of these two separate approaches had very insignificant differences due to the difference in machines precision and operating systems. Therefore, we conclude that both approaches produce identical output. Our proposed model is more optimal than the centralized approach considering the volume, confidentiality, security, and resource constraints associated with data.

## Limitations and Future Direction

There are several limitations in the current study, which will be addressed in future studies. We did not concern about the re-identification attack; we only secured the intercommunications between local and remote nodes. Our study worked with FNC datasets; however, we could study other image modalities in our subsequent studies. Moreover, we did not present many details related to the site parameter distributions as we had no accurate knowledge about the probability distribution of site parameters to compare. We want to add differential privacy and study the effects of site parameter distribution in more detail in our future studies. Moreover, in near future this algorithm will be intrigated with ENIGMA HALFpipe (30).

## Conclusion

The proposed novel model showed that decentralized algorithms could achieve identical results as their centralized counterpart. Also, the decentralized approaches solve many challenges associated with data sharing connecting the whole world. This study encouraged future researchers to contribute to making new decentralized algorithms, which will help us study all the data scattered across the world and produce beneficiary outcomes.

## Data Availability Statement

The original contributions presented in the study are included in the article/supplementary material, further inquiries can be directed to the corresponding authors.

## Author Contributions

BB, VV, VC, and FH: planned the whole project. BB and VV: responsible for conducting full research, writing manuscript, data analysis, and designing the algorithm. VC and VV: result analysis and manuscript revision. VC, VV, and FH: data analysis and result analysis. JN and HH: data collection and processing. All authors contributed to the article and approved the submitted version.

## Funding

Multiple funds supported this project from the authors VC and FH. We want to thank NIH and NSF for their grants. The grants included in this project are NSF: 2112455 and NIH: R01DA040487 and R61NS120249.

## Conflict of Interest

The authors declare that the research was conducted in the absence of any commercial or financial relationships that could be construed as a potential conflict of interest.

## Publisher's Note

All claims expressed in this article are solely those of the authors and do not necessarily represent those of their affiliated organizations, or those of the publisher, the editors and the reviewers. Any product that may be evaluated in this article, or claim that may be made by its manufacturer, is not guaranteed or endorsed by the publisher.

## Footnotes

^1^ https://en.wikipedia.org/wiki/Conjugate_prior
